# An exploratory study on low-concentration hexavalent chromium adsorption by Fe(III)-cross-linked chitosan beads

**DOI:** 10.1098/rsos.170905

**Published:** 2017-11-29

**Authors:** Yaoguo Wu, Yuanjing Zhang, Jin Qian, Xu Xin, Sihai Hu, Shuai Zhang, Jianguo Wei

**Affiliations:** Department of Applied Chemistry, Northwestern Polytechnical University, 710072 Xi'an, People's Republic of China

**Keywords:** hexavalent chromium, Fe(III)-cross-linked chitosan beads, adsorption, low concentration, pH

## Abstract

In this study, Fe(III)-cross-linked chitosan beads (Fe(III)-CBs) were synthesized and employed to explore the characteristics and primary mechanism of their hexavalent chromium (Cr(VI)) adsorption under low concentration Cr(VI) (less than 20.0 mg l^−1^) and a pH range from 2.0 to 8.0. Batch tests were conducted to determine the Cr(VI) adsorption capacity and kinetics, and the effects of pH and temperature on the adsorption under low concentration Cr(VI) and a pH range from 2.0 to 8.0. Scanning electron microscopy (SEM), Fourier-transform infrared spectroscopy and X-ray photoelectron spectroscopy were employed to explore the characteristics of Fe(III)-CBs and their Cr(VI) adsorption mechanisms. The results show that, unlike the adsorption of other absorbents, the Cr(VI) adsorption was efficient in a wide pH range from 2.0 to 6.0, and well described by the pseudo-first-order model and the Langmuir–Freundlich isotherm model. The capacity of Cr(VI) adsorption by Fe(III)-CBs was as high as 166.3 mg g^−1^ under temperature 25°C and pH 6.0. The desorption test was also carried out by 0.1 mol l^−1^ NaOH solution for Fe(III)-CBs regeneration. It was found that Fe(III)-CBs could be re-used for five adsorption–desorption cycles without significant decrease in Cr(VI) adsorption capacity. Ion exchange was confirmed between functional groups (i.e. amino group) and Cr(VI) anions (i.e. ${\text{CrO}}_{4}^{2-}$). The amino-like functional groups played a key role in Cr(VI) distribution on the Fe(III)-CBs surface; Cr(VI) adsorbed on Fe(III)-CBs was partially reduced to Cr(III) with alcoholic group served as electron donor, and then formed another rate-limiting factor. So, Fe(III)-CBs has a good prospect in purifying low concentration Cr(VI) water with a pH range from 2.0 to 6.0.

## 1. Introduction

Chromium is a common contaminant not only in industrial wastewater, derived from electroplating, metal finishing, leather tanning, steel fabrication and textile activities [1,2], but also in natural water including surface water and groundwater [3], originating from native geological environment. In nature, chromium exists in two stable oxidation states, i.e. the hexavalent (Cr(VI)) and trivalent (Cr(III)) states. The former is known to be much more toxic than the latter [4,5]. Cr(VI) is detrimental to the health when its concentration in drinking water exceeds 0.05 mg l^−1^ [6]. Therefore, it is mandatory to remove Cr(VI) anions before water supply [7]. Numerous treatment processes, including chemical precipitation, biological reduction, ion exchange and adsorption, have been studied extensively for Cr(VI) removal [8–10]. Among them, adsorption has been recognized as an economical and effective technique, and much attention has been paid to the development of cost-effective and efficient adsorbents [11–13]. Various adsorbents, such as active carbon [14], silica-gel [15], raw clays [16] and hydrotalcite [17], have been used for Cr(VI) adsorption. However, these adsorbents focused on treating industrial wastewater; although they had high adsorption capacities under acid pH, the treated water still had Cr(VI) anion, whose concentration was not low as expected, limiting their broad application. To overcome this, further treatment is needed. Meanwhile, in natural surface water and groundwater, where Cr(VI) is in low concentration and pH closes to neutrality, the Cr(VI) also needs to be treated [3]. Up to now, few studies on these have been conducted.

Chitosan is a linear cationic semisynthetic polysaccharide, which could be easily separated from seafood-processing wastes and employed as an adsorbent for pollution control [18,19]. In addition, its characteristic in adsorption was found to be superior to other adsorbents in terms of non-toxicity, hydrophilicity, biocompatibility, antibacterial activity and biodegradability [20,21]. Chitosan has also been used for Cr(VI) adsorption in water environments. Nevertheless, the capacity was found to be only 76.9 mg g^−1^ [22,23]. So enhancing the adsorption capacity of chitosan is currently one of the major tasks for Cr(VI) removal. Previous studies have indicated that binding with special metal could significantly improve the adsorption capacity of the adsorbent. The composites via linking zirconium and Fe have been employed to remove Cr(VI) in aqueous solution. And the adsorption capacities could reach 175.0 and 173.1 mg g^−1^, respectively [24,25]. The higher adsorption capacities of these metal-chitosan adsorbents imply their potential popularity in practical Cr(VI) removal. Although Cr(VI) removal at high concentrations (greater than 100 mg l^−1^) and low pH (less than 5.0) by Fe-cross-linked chitosan has been reported, no detailed study on the Cr(VI) removal by Fe-cross-linked chitosan under low concentration Cr(VI) and natural pH was carried out. Meanwhile, the mechanism of this composite to adsorb Cr(VI) deserves exploration.

In this study, Fe(III)-cross-linked chitosan beads (Fe(III)-CBs) were synthesized. The beads were employed to conduct the adsorption tests to determine the capacity, equilibrium isotherm and kinetics of the low concentration Cr(VI) (less than 20.0 mg l^−1^) adsorption under pH range from 2.0 to 8.0 pH. The surface morphology and functional groups of the beads were characterized through scanning electron microscopy (SEM) and Fourier-transform infrared (FT-IR). The oxidation states of chromium during its adsorption by Fe(III)-CBs were confirmed by X-ray photoelectron spectroscopy (XPS). Based on these data in this study, the possible mechanism of low concentration Cr(VI) adsorption by Fe(III)-CBs was proposed. These results strongly proved that Fe(III)-CBs have a good prospect in purifying low concentration Cr(VI) water with pH range 2.0–6.0.

## 2. Material and methods

### 2.1. Materials

All the reagents used in this study were of analytical grade. Chitosan (MW = 200 000, DA = 90%) was purchased from Shanghai DEMO Medical Tech. Co., Ltd (Shanghai, China). Epichlorohydrin was obtained from FUCHEN chemical reagents factory (Tianjin, China). K_2_CrO_7_, FeCl_3_·H_2_O, HCl, NaOH, Na_2_SO_4_, Na_3_PO_4_, Na_2_CO_3_, NaCl and NaNO_3_ were all purchased from Sinopharm Chemical Reagent Co., Ltd (Shanghai, China). De-ionized water was used in the preparation of all solutions. The stock solution with 100.0 mg l^−1^ Cr(VI) was prepared by dissolving 0.2848 g K_2_Cr_2_O_7_ in 1 l de-ionized water. The studied water with different Cr(VI) levels was obtained by diluting the stock K_2_Cr_2_O_7_ standard solution.

### 2.2. Methods

#### 2.2.1. Preparation of Fe(III)-CBs

The Fe(III)-CBs were prepared following the method described by Dos Santos *et al*. [26] and Qi *et al.* [27]. The main steps were as follows: Chitosan powder (2.0 g) was dissolved in 0.1 mol l^−1^ FeCl_3_ aqueous solution (100 ml), followed by stirring the solution at room temperature for 4 h. Subsequently, the Chitosan-FeCl_3_ solution was added into NaOH solution (200 ml, 10%, w/v) dropwise using a syringe to make the beads. Then the beads were separated from NaOH solution and washed with de-ionized water. Afterwards, the beads were added into de-ionized water (100 ml) before pure epichlorohydrin solution (4 ml) was added. The pH of the reaction system was adjusted to 6.0 using 0.1 M HCl or 0.1 M NaOH and kept at 70°C for a further 3 h. The cross-linked beads were dried in an oven at 60°C after the filtration. Finally, the products were treated with HCl solution (0.01 mol l^−1^) for another 3 h to remove the iron compounds on the surface and protonate the cross-linked beads.

#### 2.2.2. Characterization of Fe(III)-CBs

Surface morphology of synthesized Fe(III)-CBs was observed by SEM with an FEI Quanta 200 FEG field emission scanning electron microscope at an accelerating voltage of 20 kV. The surfaces of samples were gold coated before analysis. FT-IR spectra of Fe(III)-CBs before and after sorption were recorded at room temperature using a Bruker TENSOR 27 FT-IR spectrometer in the range of 4000–400 cm^−1^. The samples were pre-treated according to the KBr pellet method. XPS analysis was conducted before and after Cr(VI) adsorption by a Kratos Axis Ultra DLD spectrometer. All the binding energies were referenced to the C 1s peak at 284.8 eV from surface adventitious carbon.

#### 2.2.3. Batch adsorption test

The batch test was designed and carried out to evaluate the adsorption capacity of Cr(VI) on Fe(III)-CBs (2.0 g l^−1^ dosage). The temperature of all batch reactors was kept at 25 ± 1°C in an air-conditioned room and the reactors were well mixed by the shaker (Ningbo Jiangnan Instrument Factory, China) at 100 r.p.m. During the adsorption tests, the mixed liquor sample was withdrawn regularly to determine the concentration of Cr(VI).

To study the effect of pH on the Cr(VI) (5.0 mg l^−1^ initial Cr(VI) concentration) adsorption on Fe(III)-CBs, the initial pH values of the solution were adjusted to 2.0–8.0 by 0.1 mol l^−1^ HCl or 0.1 M NaOH solution. Only the optimum pH would be used in the further study.

A measure of 0.2 g Fe(III)-CBs was dosed to five batch reactors containing 100 ml of Cr(VI) solution but with different concentrations (2.5, 5.0, 10.0, 15.0, 20.0 mg l^−1^) to research the effect of Cr(VI) initial concentration for kinetic studies and variable for isotherm studies.

The influence of competing anions (${\text{SO}}_{4}^{2-}$, ${\text{CO}}_{3}^{2-}$, Cl^−^, ${\text{NO}}_{3}^{-}$ and ${\text{PO}}_{4}^{3-}$) with varying the concentration at 0.01, 0025, 0.05, 0.1 and 0.5 mol l^−1^ on the adsorption process was studied with 5.0 mg l^−1^ initial Cr(VI) concentration.

For desorption and regeneration, the Fe(III)-CBs were subjected to successive adsorption–desorption cycle tests. After the test equilibration with the initial Cr(VI) concentration of 5.0 mg l^−1^ as mentioned above, the adsorbent was separated from the solution and washed well. Thereafter, desorption tests were performed by keeping 50 ml 0.1 M NaOH solution in contact with the spent adsorbent. At the end of each cycle, the Fe(III)-CBs were washed several times with de-ionized water to remove excess base from the adsorbent surface. The reusability of Fe(III)-CBs for Cr(VI) adsorption was evaluated by conducting five cycles.

The Cr(VI) analysis was carried out spectrophotometrically at 540 nm in a spectrophotometer (Spectrovision WFJ7200, Shanghai, China), using diphenylcarbazide as the complexing agent. The amount of Cr(VI) adsorption *q*_e_ (mg g^−1^) was calculated from the equation as follows:

2.1 $$q_{e}=\frac{(C_{0}-C_{e})V}{W},$$

where *C*_0_ is the initial Cr(VI) concentration (mg l^−1^), *C*_e_ is the instantaneous Cr(VI) concentration (mg l^−1^) during the test, *V* is the volume of the aqueous phase (l) in the batch reactor and *W* is the weight of the adsorbent used (g).

The adsorption removal rate (*R*) of Cr(VI) was calculated as follows:

2.2 $$R=\frac{\left(C_{0}-C_{e}\right)}{C_{0}}\times 100\%.$$

Langmuir, Freundlich and Langmuir–Freundlich isotherms [28,29] were used for the mathematical description of the adsorption equilibrium of Cr(VI) ions on Fe(III)-CBs (table 1). The operating parameters were *T* = 25 ± 1°C, pH = 6.0, time = 200 min. The kinetic data were analysed using the pseudo-first-order, pseudo-second-order and intraparticle diffusion models (table 2) [30].

**Table 1.** Langmuir, Freundlich and Langmuir–Freundlich isotherm models and their parameters for Cr(VI) adsorption on Fe(III)-CBs (the adsorbent dosage 2.0 g l^−1^, the temperature 25 ± 1°C, the contact time 200 min). *q*_m_ is the maximum adsorption capacity (mg g^−1^); *K*_L_ is the Langmuir constant (l mg^−1^) related to the energy of adsorption. *K*_F_ is the Freundlich constant (mg l^−1^); *1/n* is the heterogeneity parameter. *K*_L-F_ is the Langmuir–Freundlich constant (l mg^−1^) related to the energy of adsorption; *c* is the heterogeneity parameter, which indicates relatively heterogeneous distribution of binding sites, while *c* is close to zero.

isotherm	equation	parameters				
	$q_{e}=\frac{q_{m}K_{L}C_{e}}{1+K_{L}C_{e}}$					
Langmuir		*q*_m_ (mg g^−1^)	*K*_L_ (l mg^−1^)		*R^2^*	*χ^2^*
		59.05	2.50		0.986	1.175
Freundlich	$q_{e}=K_{F}C^{1/n}$	*K*_F_ (mg l^−1^)		*1/n*	*R^2^*	*χ^2^*
		48.58		0.62	0.996	1.164
	qe=qmKL−FCecAA1+KL−FCec					
Langmuir–Freundlich		*q*_m_	*K*_L − F_ (l mg^−1^)	*c*	*R^2^*	*χ^2^*
		166.28	0.388	0.71	0.996	0.771

**Table 2.** Kinetic models for Cr(VI) adsorption on Fe(III)-CBs.

model	equation	parameters
pseudo-first-order kinetic model	$\ln(q_{t}-q_{e})=\ln q_{e}-k_{1}t$	*q*_e_ and *q_t_* denote the amount of Cr(VI) adsorbed at equilibrium and at time *t* (mg g^−1^), respectively; *k*_1_ is the rate constant of the model (min^−1^)
pseudo-second-order kinetic model	$\frac{t}{q_{t}}=\frac{1}{k_{2}{q_{e}}^{2}}+\frac{t}{q_{e}}$	*k*_2_ is the rate constant of pseudo-second-order model (mg g^−1^ min^−1^)
intraparticle diffusion model	$q_{t}=k_{p}t^{1/2}+c$	*k*_p_ is the intraparticle diffusion constant (mg g^−1^ min^−1/2^) and *c* (mg g^−1^) is the constant that give an idea about the effect of boundary layer thickness

## 3. Result and discussion

### 3.1. Characterization of synthesized Fe(III)-CBs

**Figure 1.**
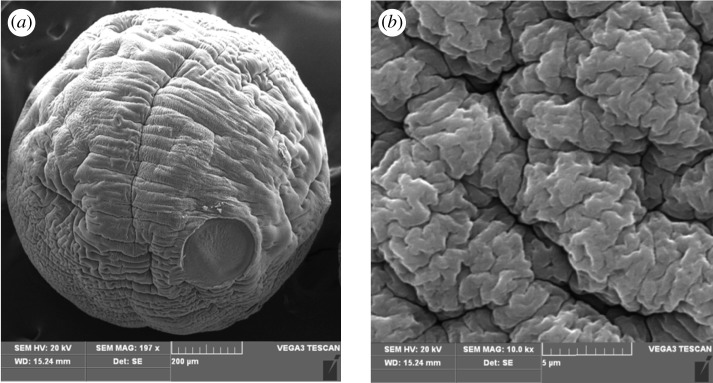
SEM images of surface of Fe(III)-CBs at low (*a*) and high magnification (*b*).

The SEM images of the Fe(III)-CBs surface are shown in figure 1. The beads appeared in the well-shaped spherical form with relatively rough and non-porous surface. The diameter of the spheres was approximately 1 mm. These findings were in agreement with the fact that beads were prepared with the suspension in aqueous solution and subsequent cross-linking [26]. Figure 1 also illustrates the existence of folds on the surface. These folds provide a large specific area and numerous active sites distributed on the surface, offering favourable conditions for Cr(VI) adsorption.

**Figure 2.**
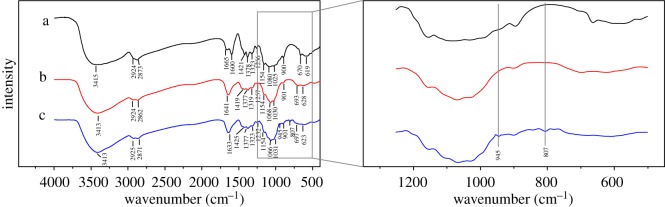
FT-IR spectra of chitosan (a), Fe(III)-CBs (b) and chromium-adsorbed Fe(III)-CBs (c).

The FT-IR spectra of chitosan and Fe(III)-CBs are presented in figure 2. For chitosan, the overlapped peak at 3415 cm^−1^ corresponded to ─OH and ─NH_2_ stretching vibrations. Two adsorption bands at 2924 and 2873 cm^−1^ were assigned to asymmetric and symmetric vibration of ─CH_2_ groups, respectively, and the adsorption peak at 1600 cm^−1^ was attributed to the characteristic peak of ─NH_2_. The spectra of Fe(III)-CBs confirm the presence of both ─NH_2_ and ─OH groups. It can be seen that the spectra had two significant shifts. The first shift of peak was at 1665–1600 cm^−1^. This shift in ─NH_2_-bending mode reflects the interaction between Fe^3+^ ion and ─NH_2_ group. The second band at 1080 cm^−1^ corresponding to primary ─OH stretching was shifted to 1068 cm^−1^, indicating the interaction between Fe^3+^ ion and ─OH group due to hydrogen bonding [6]. These depress hydrogen bonding to other ions, but promote the chitosan to be more positive. Thus, Fe(III)-CBs should have larger potential for Cr(VI) adsorption.

### 3.2. Characteristics of Cr(VI) adsorption by Fe(III)-CBs

#### 3.2.1. Effect of initial pH of the solution on Cr(VI) adsorption

**Figure 3.**
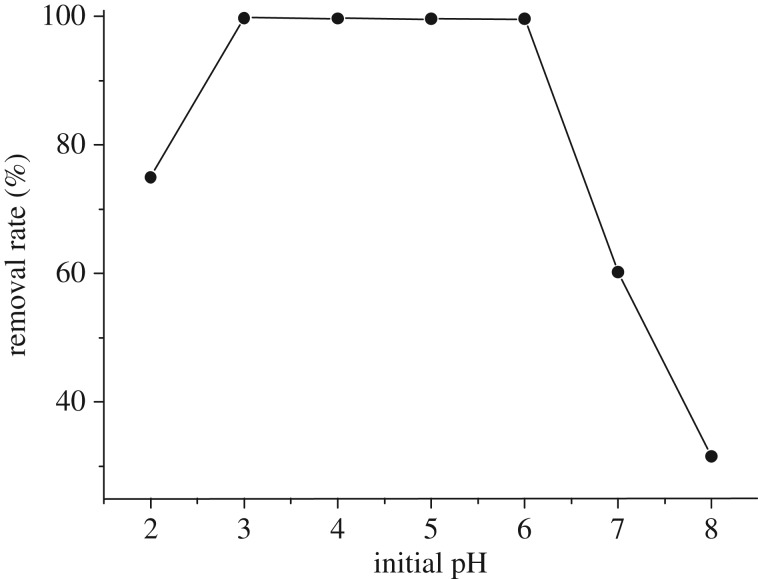
Effect of initial pH on the Cr(VI) adsorption.

The pH could significantly impact the adsorption process, because it affects not only the surface charge of the adsorbent and the degree of ionization, but also the species of adsorbate [6,31,32]. In this study, the effect of initial pH (from 2.0 to 8.0) on the adsorption process was investigated and the results are shown in figure 3.

The Cr(VI) adsorption removal rate strongly depended on the initial pH (figure 3). After 200 min reaction, Cr(VI) adsorption rate increased from 74.9 to 99.8% with pH increasing from 2.0 to 3.0, but kept similar between pH 3.0 and 6.0, finally decreased from 99.36 to 31.61% with pH constantly increasing from 6.0 to 8.0. Therefore, the optimal pH for Cr(VI) adsorption by Fe(III)-CBs ranged from 3.0 to 6.0. Similar pH-dependent trend was also found in Cr(VI) adsorption by magnetic chitosan nanoparticles [33] and protonated cross-linked chitosan [34]. In this work, the initial pH may exert effect on Cr(VI) adsorption via two facets: the surface characteristics of Fe(III)-CBs and the species of Cr(VI). The protonation by hydrogen ions (H^+^) presented in the amino groups (−NH_2_) of Fe(III)-CBs in acid solutions is depressed under basic conditions [6]. Cr(VI) exists predominantly in the form of HCrO_4_^N^ as the pH ranges from 3.0 to 6.0. The form of HCrO_4_^−^ shifts to H_2_CrO_4_ with pH lower 3.0 and shifts to ${\text{CrO}}_{4}^{2-}$ with pH higher than 6.0 [3]. Both of them worked and resulted in the characteristics of the initial pH effects, and concluded that Fe(III)-CBs adsorbed Cr(VI) as ${\text{HCrO}}_{4}^{-}$ form. The optimal pH value for Fe(III)-CBs is approximately 6.0, which is higher than others (table 3). Especially, pH is a restrictive factor for Cr(VI) absorbent and it could be overcome.

**Table 3.** Comparison of adsorption capacities of Fe(III)-CBs with other adsorbents.

adsorbent	*q*_max_/(mg g^−1^)	pH	initial concentration (mg l^−1^)	ref.
Fe(III)-CBs	166.3	6.0	20.0	this study
γ-Fe_2_O_3_-chitosan beads	106.5	5.0	1000	[32]
magnetic chitosan nanoparticles	55.8	3.0	180	[33]
composite chitosan biosorbent	153.8	2.0	400	[18]
ethylenediamine-modified cross-linked magnetic chitosan resin	51.8	2.0	100	[35]
cross-linked chitosan-Fe(III) complex	148.4	4.8	380	[36]
metal ion imprinted chitosan resin	76.9	5.5	100	[37]
Fe^0^ nanorods modified with chitosan	118.8	5.0	200	[15]
zirconium cross-linked chitosan composite	175.0	5.0	300	[25]

#### 3.2.2. Adsorption capacity and isotherms by Fe(III)-CBs

**Figure 4.**
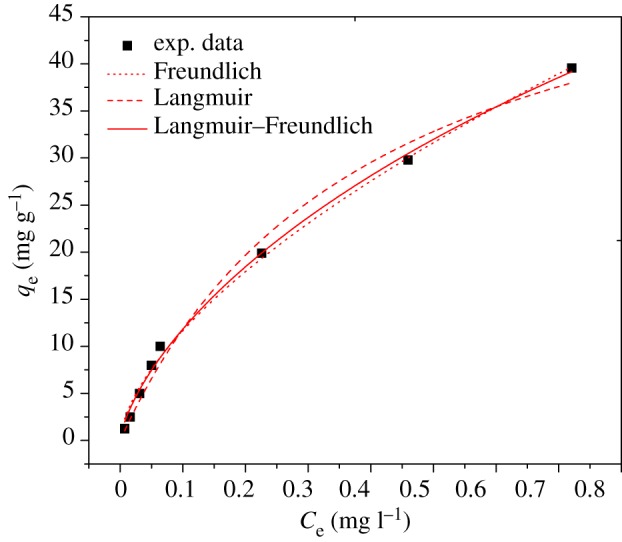
Equilibrium adsorption isotherms plot for Cr(VI) adsorption.

To examine the capacity of Cr(VI) adsorption by Fe(III)-CBs, the batch tests were performed at initial Cr(VI) concentrations, i.e. 2.5, 5.0, 10.0, 15.0 and 20.0 mg l^−1^(figure 4).

All the parameters as well as the regression coefficients of Langmuir, Freundlich and Langmuir–Freundlich models are obtained and presented in table 1. As the coefficients of correlation (*R*^2^) were close to each other (table 1), *χ*^2^ analysis was carried out to identify the suitable isotherm for the adsorption of Cr(VI). The *χ*^2^ was analysed according to the following equation:

3.1 $$\chi^{2}=\sum \frac{{\left(q_{e}-q_{m}\right)}^{2}}{q_{m}},$$

where *q*_e_ is the quantity of Cr(VI) adsorbed at the equilibrium (mg g^−1^), *q*_m_ is the maximum adsorption capacity based on the model prediction (mg g^−1^). It can be found that the Langmuir–Freundlich isotherm fitted best with the experimental data, i.e. the *R*^2^ values were higher, and *χ*^2^ values were lower. And these results (table 1) suggested that the active sites on the surface of Fe(III)-CBs are not homogeneous [38], and the Langmuir–Freundlich isotherm model is the best in the present study.

Based on the Langmuir–Freundlich isotherm model, Cr(VI) adsorption capacity of Fe(III)-CBs was determined to be 166.3 mg g^−1^ under the present conditions. As mentioned above, chitosan and its modified derivatives were characterized as great potential for Cr(VI) adsorption. The adsorption capacities of Fe(III)-CBs and other adsorbents are listed in table 3. The highest Cr(VI) removal capacity could be achieved by Fe(III)-CBs, probably attributed to its own surface characteristics (see §3.1). In view of its high adsorption capacity, Fe(III)-CBs can be recommended as an efficient alternative for the low concentration Cr(VI) adsorption.

#### 3.2.3. Adsorption kinetics

**Figure 5.**
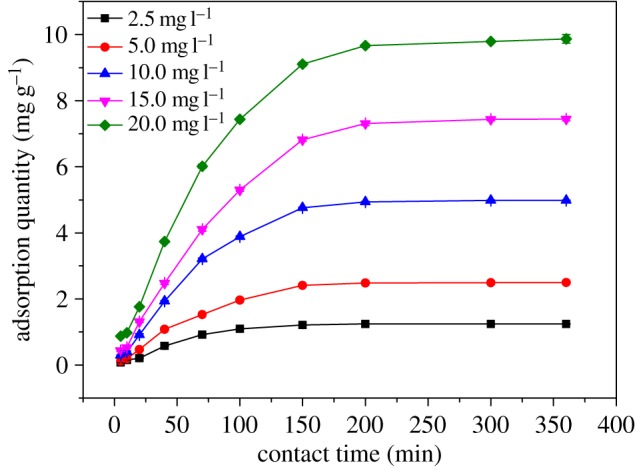
Effect of initial Cr(VI) concentration on adsorption capacity on Fe(III)-CBs.

The effect of initial Cr(VI) concentration on adsorption capacity on Fe(III)-CBs is presented in figure 5. Figure 5 shows that the adsorbed amount of Cr(VI) increased rapidly in the first 100 min, contributing to approximately 80% of the ultimate adsorption capacity. And the adsorption equilibrium reached within 200 min. Comparatively, the required times to achieve equilibrium for the zirconium–chitosan composite and γ-Fe_2_O_3_−chitosan beads were approximately 300 and 360 min [39] respectively; as a result, the Fe(III)-CBs shows better performance in Cr(VI) adsorption.

To determine the adsorption kinetics, the models in table 2 were applied here to show whether they could match the experimental data (table 4). Table 4 shows that the coefficient of correlation (*R*^2^) was much higher, and the theoretical *q*_e*,*cal_ value was closer to the experimental *q*_e,exp_ value in the case of the pseudo-first-order kinetic model. Therefore, the pseudo-first-order model fitted the experimental data better than the pseudo-second-order model. This is in accordance with the Cr(VI) adsorption on Fe(III) hydroxide-loaded sugar beet pulp [11]. Because the pseudo-first-order model is based on the adsorption rate being proportional to the difference between the equilibrium adsorption capacity and the adsorbed amount [20,40], while the adsorbed amount is controlled by Cr(VI) diffusion to the adsorbent, Fe(III)-CBs have the higher kinetic constant *k*_1_, indicating that this adsorbent is easy to close for Cr(VI).

**Table 4.** Kinetic parameters for Cr(VI) adsorption on Fe(III)-CBs.

		pseudo-first-order model			pseudo-second-order model		
initial Cr(VI) concentration (mg l^−1^)	$q_{e,\exp}$ (mg g^−1^)	$q_{e,\mathrm{cal}}$ (mg g^−1^)	*k*_1_ (min^−1^)	*R*^2^	$q_{e,\mathrm{cal}}$ (mg g^−1^)	*k*_2_ (mg g^−1^ min^−1^)	*R*^2^
2.5	1.24	1.35	0.023	0.989	2.67	0.0024	0.809
5.0	2.49	2.78	0.019	0.987	8.76	0.0028	0.356
10.0	4.99	5.15	0.018	0.989	17.73	0.0027	0.458
15.0	7.44	8.00	0.017	0.996	25.02	0.0026	0.731
20.0	9.97	11.68	0.016	0.997	24.22	0.0043	0.883

#### 3.2.4. Effect of coexisting anions

During the adsorption process, the coexisting anions may compete with Cr(VI) for active sites on the adsorbent, and thus negatively affect Cr(VI) adsorption performance. This competition is the result of electrostatic interaction and ligand exchange reaction [24,31]. So the effects of several common anions in natural water [17], such as ${\text{HCO}}_{3}^{-}$, ${\text{H}}_{2}{\text{PO}}_{4}^{-}$, ${\text{SO}}_{4}^{2-}$, Cl^−^ and ${\text{NO}}_{3}^{-}$ on Cr(VI) adsorption, were evaluated.

**Figure 6.**
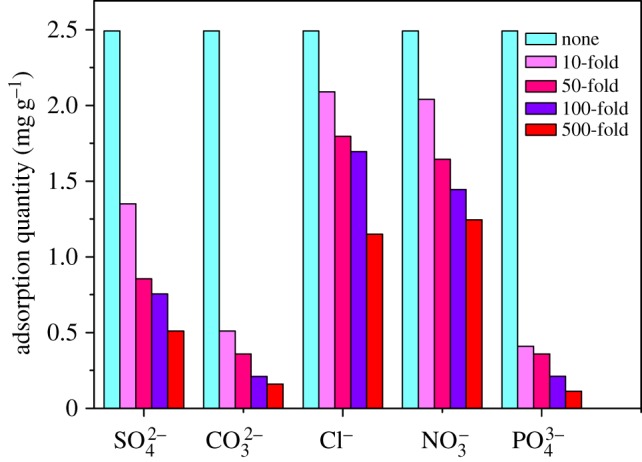
Effect of coexisting anions on Cr(VI) adsorption.

The results show that all the anions selected here could depress the Cr(VI) adsorption capacity (figure 6). Nevertheless, the extent of depression by each anion is different and ranked as ${\text{HCO}}_{3}^{-}$≈ ${\text{H}}_{2}{\text{PO}}_{4}^{-}$ > ${\text{SO}}_{4}^{2-}$ > Cl^−^ ≈ ${\text{NO}}_{3}^{-}$. When ${\text{HCO}}_{3}^{-}$, ${\text{H}}_{2}{\text{PO}}_{4}^{-}$, ${\text{SO}}_{4}^{2-}$, Cl^−^ or ${\text{NO}}_{3}^{-}$ presented with 10-fold of Cr(VI) concentration, the equilibrium Cr(VI) adsorption capacities were lowered by 16.1%, 18.1%, 45.8%, 79.5% and 83.5% compared with that without any coexisting anion. And increasing the above coexisting anion concentration to 50-fold of Cr(VI) concentration, the adsorption capacities were further decreased by 6.4%, 4.5%, 29.7%, 46.2% and 50.0%, respectively. Compared with the influence of Cl^−^ and ${\text{NO}}_{3}^{-}$, ${\text{HCO}}_{3}^{-}$ and ${\text{H}}_{2}{\text{PO}}_{4}^{-}$ may lead to stronger competition with Cr(VI) in the activated sites of Fe(III)-CBs and depress the Cr(VI) removal to a larger extent.

#### 3.2.5. Regeneration and reuse of Fe(III)-CBs

Reusability is one of the most important parameters to assess the applicability of an adsorbent in practice. Based on the results above, 0.1 mol l^−1^ Na_2_SO_4_, Na_2_CO_3_, Na_3_PO_4_, NaOH and EDTA were used as desorption solution. The desorption removal rates were 2.54%, 18.40%, 48.00%, 65.80% and 0.58% for the five desorption solutions, respectively. Among them, NaOH solution desorbing Cr(VI) was most effective [25]. This result was consistent with the results of the pH effect above, indicating that electrostatic interaction between the adsorbent and Cr(VI) plays a significant role in the adsorption. So, in this view, adjusting pH is a practical way to regenerate Fe(III)-CBs. So NaOH solution was used as desorption solution for the regeneration and reuse of Fe(III)-CBs. The tests were conducted with 0.01, 0.05, 0.1, 0.5 or 1.0 mol l^−1^ NaOH concentration to determine its optimal concentration. Their results, lower than 0.1 mol l^−1^ of NaOH solution, more or less the desorption rate; and higher than 0.1 mol l^−1^, the desorption rate, hardly varied and kept close to 70%. So, the optimal concentration is 0.1 mol l^−1^ for NaOH solution as desorption solution.

**Figure 7.**
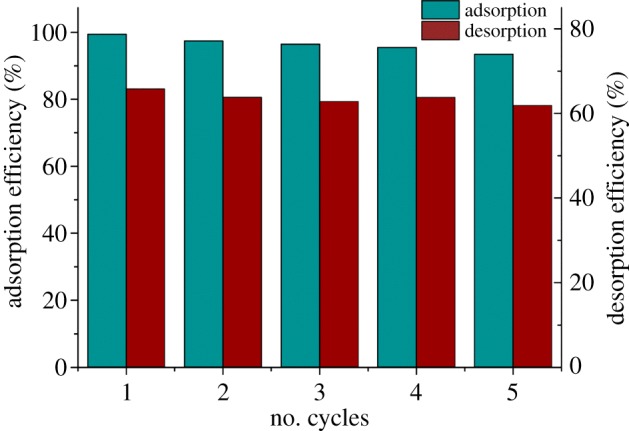
Cr(VI) adsorption–desorption behaviours on Fe(III)-CBs.

The tests were still conducted to evaluate the possibility of Fe(III)-CBs reuse for Cr(VI) adsorption undergoing five cycles (figure 7). Their data proved that the adsorption capacity for Cr(VI) was not significantly changed with the cycle number and more than 93% of initial adsorption capacity was maintained after five adsorption/desorption/regeneration cycles.

### 3.3. Proposed process and mechanism

**Figure 8.**
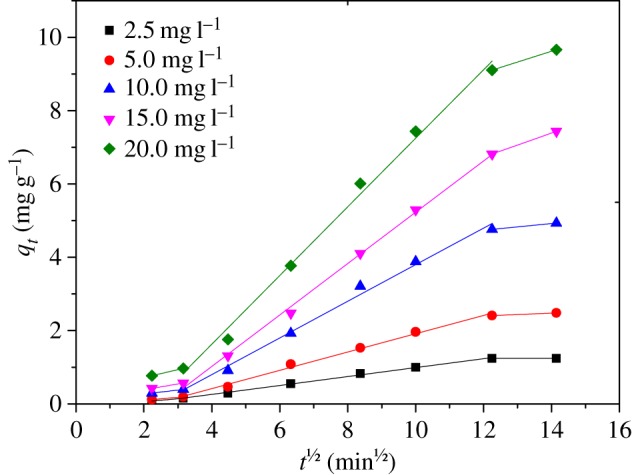
Plots of *q_t_* versus *t*^1/2^ for the adsorption of Cr(VI) ranging from 2.5 to 20.0 mg l^−1^ (the Fe(III)-CBs dosage 2.0 g l^−1^, the temperature 25 ± 1°C).

Generally, the adsorption process can be divided into three steps: (i) external surface adsorption, (ii) intraparticle diffusion and (iii) the final equilibrium [20,41]. Among them, the intraparticle diffusion is generally regarded as the rate-limiting step [42]. Based on the intraparticle diffusion model, when the plot of *q_t_* versus *t*^1/2^ gives a straight line and passes through the origin, the adsorption process is considered to be controlled only by intraparticle diffusion. When the plot of *q_t_* versus *t*^1/2^ exhibits the multilinear plot, the adsorption process should be controlled by both intraparticle diffusion step and other steps [20,42–45]. In this work, the value of *k*_p_ became higher as initial Cr(VI) concentration increased (table 5), suggesting the concentration of Cr(VI) has significant influence on the intraparticle diffusion. Meanwhile, the value of *R^2^* higher than 0.99 revealed that the intraparticle diffusion plays a vital role in the adsorption process. However, the plot of *q_t_* versus *t*^1/2^ shown in figure 8 illustrates that the intraparticle diffusion was not the only rate-limiting step.

**Table 5.** Parameters for Cr(VI) adsorption on Fe(III)-CBs.

	parameters for intraparticle diffusion		
initial Cr(VI) concentration (mg l^−1^)	*C* (mg g^−1^)	*k*_p_ (mg g^−1^ min^0.5^)	*R*^2^
2.5	−0.228	0.122	0.996
5.0	−0.579	0.249	0.993
10.0	−1.199	0.499	0.991
15.0	−1.774	0.701	0.997
20.0	−2.386	0.964	0.994

It has been the Cr(VI) adsorption by Chitosan mainly through the interaction between several function groups (such as amino group) and Cr(VI) [6]. So, the quantities of groups and their exposure to Cr(VI) are essential for the adsorption. As shown in figure 2, the FT-IR spectrum of Cr(VI) adsorbed on Fe(III)-CBs displayed an obvious formation of new bands at 945 and 807 cm^−1^. According to the literature [24,44], these two peaks were attributed to Cr(VI)─O and Cr(III)─OH vibration. This confirms the presence of Cr(VI) on the surface of Fe(III)-CBs and demonstrates a part of adsorbed Cr(VI) was reduced to Cr(III) on the sorbent.

**Figure 9.**
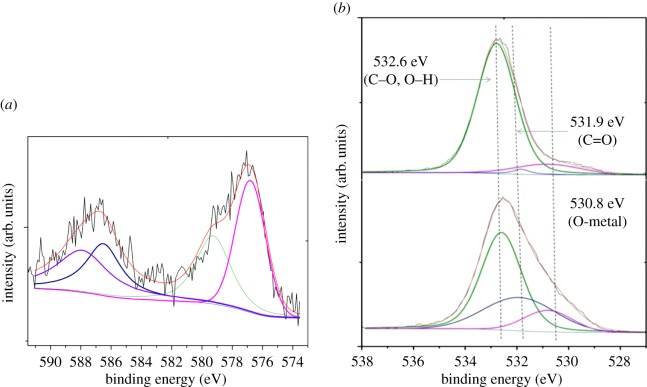
XPS spectra of Cr 2p (*a*) and O 1s (*b*) on Cr-adsorbed Fe(III)-CBs.

XPS spectra of Fe(III)-CBs before and after Cr(VI) adsorption are presented as figure 9. The photoelectron peaks for the Cr 2p_3/2_ and Cr 2p_1/2_ are located at 576.9 and 586.8 eV (figure 9*a*), respectively. The broad peak of Cr 2p_3/2_ could be fitted into two peaks at binding energies of 579.3 and 576.8 eV, which were the characteristics of Cr(VI) and Cr(III), respectively. On the other hand, the peaks at 588.0 and 586.5 eV could be attributed to Cr(VI) and Cr(III) binding energies for 2p_1/2_ orbits [24,45], manifesting both Cr(VI) and Cr(III) coexisted on the surface of the Fe(III)-CBs after adsorption. The photoelectron peaks for O 1s could be fitted by three peaks at binding energies of 530.8, 531.9 and 532.6 eV (figure 9*b*), respectively. The dominant peak located at 532.6 eV assigned to C─O, O═H or bound H_2_O, and the peak at around 530.8 suggested the existence of Fe(III)═O. As the adsorption proceeded, the peak that appeared at 531.9 eV showed an obvious increasing intensity, indicating that the bond of C═O enhanced significantly after adsorption of Cr(VI). The C═O band was attributed to the oxidation of alcoholic group (−CH_2_OH) on the molecular chain of chitosan [24]. It is therefore suggested that the alcoholic group was the electron donor in the process of the redox reaction, in which Cr(VI) was reduced to Cr(III), while the CH_2_OH was oxidized to C═O. When the pH ranges from 3.0 to 6.0, Cr(III) exists predominantly as Cr(OH)^2+^ [46], the bond between Cr(VI) and Fe(III)-CBs was broken due to the change of the electrostatic interaction. Furthermore, the free Cr(OH)^2+^ may be chelated with amine groups or precipitated onto the surface of Fe(III)-CBs in the hydroxide nature. The reduction of Cr(VI) to Cr(III) is the reason for not only the desorption rate, but also the adsorption rate-limiting step.

Therefore, the possible mechanism of Cr(VI) adsorbed on Fe(III)-CBs can be proposed: HCrO_4_^−^ was first adsorbed onto Fe(III)-CBs through electrostatic interaction and ligand–exchange interaction. Then, the Cr(VI) adsorbed via electrostatic interaction was partially reduced to Cr(III) with the ─CH_2_OH on the adsorbent served as the electron donor. The resulted Cr(III) was either chelated with NH_2_ on the Fe(III)-CBs or precipitated onto the surface of the adsorbent.

## 4. Conclusion

In this study, Fe(III)-CBs were synthesized and characterized by SEM, FT-IR and XPS, and applied for low concentration Cr(VI) adsorption. The efficient Cr(VI) adsorption was achieved by the composites within low concentration Cr(VI) and pH range from 2.0 to 6.0. And the adsorption process was well agreed with the Langmuir–Freundlich adsorption model and pseudo-first-order kinetic model. The maximum adsorption efficiency and capacity were 99.36% and 166.3 mg g^−1^ at pH 6.0, respectively. Both electrostatic interaction and ligand exchange reaction play important roles in the adsorption processes, and the anions including OH^−^, $\;{\text{SO}}_{4}^{2-}$, ${\text{CO}}_{3}^{2-}$ and ${\text{PO}}_{4}^{2-}$ could compete with Cr(VI) on the active adsorption sites. The beads were about 1 mm in average diameter and easy to be separated by filtration, and then regenerated and re-used effectively for the adsorption of Cr(VI). A part of Cr(VI) adsorbed on the beads was reduced to Cr(III) with the ─CH_2_OH serving as the electron donor, which reduced the toxicity of chromium. Over all, Fe(III)-CBs have the potential of practical application for the adsorption of Cr(VI) from water.

## Data accessibility

All of the data in this investigation have been reported in the paper and are freely available.

## References

[1] Ma J, Zuo-Jiang SZ, He Y, Sun Q, Wang Y, Liu W, Sun S, Chen K. 2016 A facile, versatile approach to hydroxyl-anchored metal oxides with high Cr(VI) adsorption performance in water treatment. R. Soc. open sci. 3, 160524 (doi:10.1098/rsos.160524) 28018639 10.1098/rsos.160524 PMC5180137

[2] Mohseni-Bandpi A, Kakavandi B, Kalantary RR, Azari A, Keramati A 2015 Development of a novel magnetite–chitosan composite for the removal of fluoride from drinking water: adsorption modeling and optimization. RSC Adv. 5, 73 279–73 289. (doi:10.1039/C5RA11294J)

[3] Rakhunde R, Deshpande L, Juneja HD 2015 Chemical speciation of chromium in water: a review. Crit. Rev. Environ. Sci. Technol. 42, 776–810. (doi:10.1080/10643389.2010.534029)

[4] Shi LN, Zhang X, Chen ZL 2011 Removal of chromium (VI) from wastewater using bentonite-supported nanoscale zero-valent iron. Water Res. 45, 886–892. (doi:10.1016/j.watres.2010.09.025) 20950833 10.1016/j.watres.2010.09.025

[5] Metters JP, Kadara RO, Banks CE 2012 Electroanalytical sensing of chromium(III) and (VI) utilising gold screen printed macro electrodes. Analyst 137, 896–902. (doi:10.1039/c2an16054d) 22228309 10.1039/c2an16054d

[6] Zimmermann AC, Mecabô A, Fagundes T, Rodrigues CA 2010 Adsorption of Cr(VI) using Fe-crosslinked chitosan complex (Ch-Fe). J. Hazard. Mater. 179, 192–196. (doi:10.1016/j.jhazmat.2010.02.078) 20307932 10.1016/j.jhazmat.2010.02.078

[7] Chen DK, Wang H 2015 Cr(VI) removal by combined redox reactions and adsorption using pectin-stabilized nanoscale zero-valent iron for simulated chromium contaminated water. RSC Adv. 5, 65 068–65 073. (doi:10.1039/C5RA10573K)

[8] Fang J, Gu ZM, Gang DC, Liu CX, Ilton ES, Deng BL 2007 Cr(VI) removal from aqueous solution by activated carbon coated with quaternized poly(4-vinylpyridine). Environ. Sci. Technol. 41, 4748–4753. (doi:10.1021/es061969b) 17695924 10.1021/es061969b

[9] Keshmirizadeh E, Yousefi S, Rofouei MK 2011 An investigation on the new operational parameter effective in Cr(VI) removal efficiency: a study on electrocoagulation by alternating pulse current. J. Hazard. Mater. 190, 119–124. (doi:10.1016/j.jhazmat.2011.03.010) 21531074 10.1016/j.jhazmat.2011.03.010

[10] Wang P, Lo IMC 2009 Synthesis of mesoporous magnetic gamma-Fe_2_O_3_ and its application to Cr(VI) removal from contaminated water. Water Res. 43, 3727–3734. (doi:10.1016/j.watres.2009.05.041) 19559458 10.1016/j.watres.2009.05.041

[11] Altundogan HS 2005 Cr(VI) removal from aqueous solution by iron (III) hydroxide-loaded sugar beet pulp. Process Biochem. 40, 1443–1452. (doi:10.1016/j.procbio.2004.06.027)

[12] Rao RAK, Rehman F 2010 Adsorption studies on fruits of Gular (*Ficus glomerata*): removal of Cr(VI) from synthetic wastewater. J. Hazard. Mater. 181, 405–412. (doi:10.1016/j.jhazmat.2010.05.025) 20605325 10.1016/j.jhazmat.2010.05.025

[13] Sharma YC, Srivastava V, Weng CH, Upadhyay SN 2009 Removal of Cr(VI) from wastewater by adsorption on iron nanoparticles. Can. J. Chem. Eng. 87, 921–929. (doi:10.1002/cjce.20230)

[14] Miao MS, Wang YN, Kong Q, Shu L 2015 Adsorption kinetics and optimum conditions for Cr(VI) removal by activated carbon prepared from luffa sponge. Desalin. Water Treat. 57, 7763–7772. (doi:10.1080/19443994.2015.1015453)

[15] Li XT, Han CY, Zhu WJ, Ma WH, Luo YM, Zhou Y, Yu J, Wei KX 2014 Cr(VI) Removal from aqueous by adsorption on amine-functionalized mesoporous silica prepared from silica fume. J. Chem. 11, 1–10. (doi:10.1155/2014/765856)

[16] Göde F, Öztürk N, Sert Y, Bahçeli S 2010 Adsorption of Cr(VI) from aqueous solutions onto raw and acid-activated reşadiye and hançılı clays. Spectr. Lett. 43, 68–78. (doi:10.1080/00387010903261164)

[17] Xu YF, Zhang J, Qian GR, Ren Z, Xu ZP, Wu YY, Liu Q, Qiao SZ 2010 Effective Cr(VI) removal from simulated groundwater through the hydrotalcite-derived adsorbent. Ind. Eng. Chem. Res. 49, 2752–2758. (doi:10.1021/ie901469c)

[18] Boddu VM, Krishnaiah A, Talbott JL, Smith ED, Richard H 2008 Removal of arsenic (III) and arsenic (V) from aqueous medium using chitosan-coated biosorbent. Water Res. 42, 633–642. (doi:10.1016/j.watres.2007.08.014) 17822735 10.1016/j.watres.2007.08.014

[19] Bhattacharya AK, Naiya TK, Mandal SN, Das SK 2008 Adsorption, kinetics and equilibrium studies on removal of Cr(VI) from aqueous solutions using different low-cost adsorbents. Chem. Eng. J. 137, 529–541. (doi:10.1016/j.cej.2007.05.021)

[20] Dragan ES, Apopei Loghin DF, Cocarta AI 2014 Efficient sorption of Cu^2+^ by composite chelating sorbents based on potato starch-graft-polyamidoxime embedded in chitosan beads. ACS Appl. Mat. Int. 6, 16 577–16 592. (doi:10.1021/am504480q) 10.1021/am504480q 25191990

[21] Qian L, Zhang HF 2010 Green synthesis of chitosan-based nanofibers and their applications. Green Chem. 12, 1207–1214. (doi:10.1039/b927125b)

[22] Ngah WSW, Kamari A, Fatinathan S, Ng PW 2006 Adsorption of chromium from aqueous solution using chitosan beads. Adsorption 12, 249–257. (doi:10.1007/s10450-006-0501-0)

[23] Owlad M, Aroua MK, Wan AWD, Baroutian S 2008 Removal of hexavalent chromium-contaminated water and wastewater: a review. Water Air Soil Poll. 200, 59–77. (doi:10.1007/s11270-008-9893-7)

[24] Shen CS, Chen H, Wu SS, Wen YZ, Li LN, Jiang Z, Li MC, Liu WP 2013 Highly efficient detoxification of Cr(VI) by chitosan–Fe(III) complex: process and mechanism studies. J. Hazard. Mater. 244, 689–697. (doi:10.1016/j.jhazmat.2012.10.061) 23200119 10.1016/j.jhazmat.2012.10.061

[25] Zhang LF, Xia W, Teng B, Liu X, Zhang WQ 2013 Zirconium cross-linked chitosan composite: preparation, characterization and application in adsorption of Cr(VI). Chem. Eng. J. 229, 1–8. (doi:10.1016/j.cej.2013.05.102)

[26] dos Santos HH, Demarchi CA, Rodrigues CA, Greneche JM, Nedelko N, Slawska-Waniewska A 2011 Adsorption of As(III) on chitosan-Fe-crosslinked complex (Ch-Fe). Chemosphere 82, 278–283. (doi:10.1016/j.chemosphere.2010.09.033) 20943252 10.1016/j.chemosphere.2010.09.033

[27] Qi JY, Zhang GS, Li HN 2015 Efficient removal of arsenic from water using a granular adsorbent: Fe─Mn binary oxide impregnated chitosan bead. Bioresour. Technol. 193, 243–249. (doi:10.1016/j.biortech.2015.06.102) 26141284 10.1016/j.biortech.2015.06.102

[28] Aydın YA, Aksoy ND 2009 Adsorption of chromium on chitosan: optimization, kinetics and thermodynamics. Chem. Eng. J. 151, 188–194. (doi:10.1016/j.cej.2009.02.010)

[29] Hena S 2010 Removal of chromium hexavalent ion from aqueous solutions using biopolymer chitosan coated with poly 3-methyl thiophene polymer. J. Hazard. Mater. 181, 474–479. (doi:10.1016/j.jhazmat.2010.05.037) 20627405 10.1016/j.jhazmat.2010.05.037

[30] Sağ Y, Aktay Y 2002 A comparative study for the sorption of Cu(II) ions by chitin and chitosan: application of equilibrium and mass transfer models. Sep. Sci. Technol. 37, 2801–2822. (doi:10.1081/SS-120005467)

[31] Shen CS, Shen Y, Wen YZ, Wang HY, Liu WP 2011 Fast and highly efficient removal of dyes under alkaline conditions using magnetic chitosan-Fe(III) hydrogel. Water Res. 45, 5200–5210. (doi:10.1016/j.watres.2011.07.018) 21839488 10.1016/j.watres.2011.07.018

[32] Zhu HY, Jiang R, Xiao L, Li W 2010 A novel magnetically separable γ-Fe_2_O_3_/crosslinked chitosan adsorbent: preparation, characterization and adsorption application for removal of hazardous azo dye. J. Hazard. Mater. 179, 251–257. (doi:10.1016/j.jhazmat.2010.02.087) 20334972 10.1016/j.jhazmat.2010.02.087

[33] Thinh NN, Hanh PTB, Ha LTT, Anh LN, Hoang TV, Hoang VD, Dang LH, Khoi NV, Lam TD 2013 Magnetic chitosan nanoparticles for removal of Cr(VI) from aqueous solution. Mater. Sci. Eng. C 33, 1214–1218. (doi:10.1016/j.msec.2012.12.013) 10.1016/j.msec.2012.12.013 23827563

[34] Huang RH, Yang BC, Liu Q 2013 Removal of chromium(VI) ions from aqueous solutions with protonated crosslinked chitosan. J. Appl. Polym. Sci. 129, 908–915. (doi:10.1002/app.38685)

[35] Hu XJ, Wang JS, Liu YG, Li X, Zeng GM, Bao ZL, Zeng XX, Chen AW, Long F. 2011 Adsorption of chromium (VI) by ethylenediamine-modified cross-linked magnetic chitosan resin: isotherms, kinetics and thermodynamics. J. Hazard. Mater. 185, 306–314. (doi:10.1016/j.jhazmat.2010.09.034) 20889258 10.1016/j.jhazmat.2010.09.034

[36] Demarchi CA, Debrassi A, Magro JD, Nedelko N, Ślawska-Waniewska A, Dłużewski P, Greneche JM, Rodrigues CA 2015 Adsorption of Cr(VI) on crosslinked chitosan–Fe(III) complex in fixed-bed systems. J. Water Process Eng. 7, 141–152. (doi:10.1016/j.jwpe.2015.05.003).

[37] Tianwei T, He X, Du W 2001 Adsorption model of metal ion on imprinting chitosan resin. J. Chem. Technol. Biotechnol. 76, 191–195. (doi:10.1002/jctb.358)

[38] Meenakshi S, Viswanathan N 2007 Identification of selective ion exchange resin for fluoride sorption. J. Colloid Interface Sci. 308, 438–450. (doi:10.1016/j.jcis.2006.12.032) 17258756 10.1016/j.jcis.2006.12.032

[39] Jiang YJ, Yu XY, Luo T, Jia Y, Liu JH, Huang XJ 2013 γ-Fe_2_O_3_ Nanoparticles encapsulated millimeter-sized magnetic chitosan beads for removal of Cr(VI) from water: thermodynamics, kinetics, regeneration, and uptake mechanisms. J. Chem. Eng. Data 58, 3142–3149. (doi:10.1021/je400603p)

[40] Liu Y, Liu ZC, Gao J, Dai JD, Han JA, Wang Y, Xie JM, Yan YS 2011 Selective adsorption behavior of Pb(II) by mesoporous silica SBA-15-supported Pb(II)-imprinted polymer based on surface molecularly imprinting technique. J. Hazard. Mater. 186, 197–205. (doi:10.1016/j.jhazmat.2010.10.105) 21109351 10.1016/j.jhazmat.2010.10.105

[41] Ma J et al. 2012 Enhanced adsorptive removal of methyl orange and methylene blue from aqueous solution by alkali-activated multiwalled carbon nanotubes. ACS Appl. Mat. Inter. 4, 5749–5760. (doi:10.1021/am301053m) 10.1021/am301053m 23062571

[42] Yan LG, Qin LL, Yu HQ, Li S, Shan RR, Du B 2015 Adsorption of acid dyes from aqueous solution by CTMAB modified bentonite: kinetic and isotherm modeling. J. Mol. Liq. 211, 1074–1081. (doi:10.1016/j.molliq.2015.08.032)

[43] Shaaban AF, Fadel DA, Mahmoud AA, Elkomy MA, Elbahy SM 2014 Synthesis of a new chelating resin bearing amidoxime group for adsorption of Cu(II), Ni(II) and Pb(II) by batch and fixed-bed column methods. J. Environ. Chem. Eng. 2, 632–641. (doi:10.1016/j.jece.2013.11.001)

[44] Das SK, Manabendra M, Guha AK 2008 Interaction of chromium with resistant strain *Aspergillus versicolor*: investigation with atomic force microscopy and other physical studies. Langmuir. 24, 8643–8650. (doi:10.1021/la800958u) 18598062 10.1021/la800958u

[45] Park D, Yun YS, Park JM 2008 XAS and XPS studies on chromium-binding groups of biomaterial during Cr(VI) biosorption. J. Colloid. Interf. Sci. 317, 54–61. (doi:10.1016/j.jcis.2007.09.049) 10.1016/j.jcis.2007.09.049 17935729

[46] Yun YS, Park D, Park JM, Volesky B 2001 Biosorption of trivalent chromium on the brown seaweed biomass. Environ. Sci. Technol. 35, 4353–4358. (doi:10.1021/es010866k) 11718356 10.1021/es010866k

